# Digital fatigue and job burnout among nurses: serial indirect associations through fear of missing out and perceived stress

**DOI:** 10.3389/fpsyg.2026.1947843

**Published:** 2026-10-28

**Authors:** Zhuliu Gong, Yi Guo

**Affiliations:** School of Journalism and Communication, Chongqing University, Chongqing, China

**Keywords:** digital fatigue, fear of missing out, job burnout, nurses, perceived stress

## Abstract

**Background:**

Digital systems are integral to nursing practice, but sustained digital engagement may be accompanied by fatigue. The roles of general fear of missing out (FoMO) and perceived stress in the association between digital fatigue and nurses’ job burnout remain unclear. Guided by conservation of resources theory, this study examined the separate and serial indirect associations through FoMO and perceived stress.

**Method:**

A cross-sectional survey included 554 clinical nurses recruited by convenience sampling from five tertiary Grade A hospitals in Hunan, China, between January and May 2026. Self-report measures assessed the four constructs. Structural equation modeling with bootstrap confidence intervals examined the proposed associations.

**Results:**

Digital fatigue remained positively associated with job burnout when FoMO and perceived stress were included in the model. Positive indirect associations were identified through each variable separately and through FoMO followed by perceived stress (effect = 0.041, 95% CI [0.021, 0.066]).

**Conclusion:**

Digital fatigue was positively associated with nurses’ job burnout, both directly and indirectly through general FoMO and perceived stress. Hospital managers could assess digital fatigue, stress, and burnout when reviewing digital workflows. Clearer expectations for responding to work messages and protected recovery time warrant prospective evaluation, including effects on staff wellbeing, workflow, and patient safety.

## 1 Introduction

The digital transformation of health care has substantially reshaped nursing practice over the past three decades (Li and Zhong, 2026). As frontline staff, nurses must not only learn to operate smart devices but also develop competence in collecting, recording, and processing data (Kılıç, 2016). These technologies improve information access and care coordination. Their sustained use, however, increases documentation demands, interruptions, cognitive load, and connectivity beyond scheduled working hours (Alobayli et al., 2023; Kräft et al., 2024; Schlicht et al., 2025; Wirth et al., 2024; Wu et al., 2024). Because these systems are indispensable to time-sensitive clinical work, nurses cannot simply reduce their digital engagement when it becomes exhausting. Digital fatigue refers to the cognitive, emotional, and physical depletion associated with sustained interaction with digital technologies (Tutar and Mutlu, 2024). Nurses may thus feel exhausted by intensive digital work yet remain reluctant to disconnect, for fear of missing important clinical information, work-related interactions, or urgent requests. This technology-related depletion is an occupational health concern. Among nurses, it has been associated with occupational stress and reduced job performance (Demir et al., 2025), and digital technology stress has been linked to emotional exhaustion and occupational burnout in this population (Li and Zhong, 2026). Studies in other sectors report a similar association with elevated burnout (Hadjri et al., 2025). Job burnout is a work-related syndrome characterized by exhaustion, mental distance from work or cynicism, and reduced professional efficacy (Maslach and Leiter, 2016; World Health Organization, 2019). It is prevalent in the global nursing workforce and is associated with turnover intentions, poorer care quality, and patient-safety risks (Ge et al., 2023; Getie et al., 2025; Li et al., 2024b). Understanding whether and how digital fatigue is associated with job burnout therefore matters for both nurse well-being and the quality of clinical care.

Chinese tertiary hospitals provide a particularly relevant setting for examining this relationship. National smart-hospital policy has incorporated digital systems into routine clinical workflows (National Health Commission of the People’s Republic of China, 2020). In a multicentre study of 1,612 Chinese nurses, 97.33% were required to use information and communication technologies (ICT) at work, and 43.30% used them for more than 5 hours per day (Guo et al., 2025). Burnout-related experiences are also common. A national multistage survey found that 50.70% of Chinese nurses reported moderate-to-high emotional exhaustion and 32.80% reported moderate-to-high depersonalization (Li et al., 2024a). Required digital engagement and a substantial burnout burden thus coexist in this setting, yet the association between digital fatigue and job burnout among Chinese nurses has rarely been examined.

Existing research offers only a partial account of this problem. Studies of technostress, electronic health record burden, and ICT demands have shown that documentation workload, usability problems, technological complexity, interruptions, and after-hours electronic work are associated with stress and job burnout among health professionals (Alobayli et al., 2023; Guo et al., 2025; Wirth et al., 2024; Wu et al., 2024). These studies focus mainly on digital working conditions or on employees’ appraisals of those conditions. Explanations of nurses’ stress, job performance, and turnover intention still rely largely on conventional factors, such as organizational justice, organizational climate, and occupational stress (Song et al., 2024; Yang et al., 2021). New stressors arising from digitalization, digital fatigue in particular, have received limited attention, especially in the Chinese context. Digital fatigue shifts attention to the depletion experienced during sustained digital engagement (Tutar and Mutlu, 2024). What remains unclear is the psychological process that connects this depletion with nurses’ broader relationship with work: why digitally fatigued nurses may remain psychologically connected to digital work, and how this continuing concern becomes associated with broader stress appraisal and job burnout.

Fear of missing out (FoMO) and perceived stress may jointly explain this process. FoMO refers to the apprehension that one might miss rewarding experiences, together with the desire to stay connected with what others are doing (Przybylski et al., 2013; Tandon et al., 2021). When digital engagement is required rather than optional, depleted nurses may remain vigilant about missing work-relevant information and interactions. Fatigue and connectivity concern can then persist together. Consistent with this reasoning, workplace and information-related FoMO have been associated with after-hours ICT use, impaired psychological detachment, perceived stress, and exhaustion (Marsh et al., 2024; Reimann et al., 2024). General FoMO has also been positively associated with job burnout among nurses (Qutishat and Al Sabei, 2024). Although FoMO is usually treated as an antecedent of sustained digital use, longitudinal evidence suggests that FoMO and fatigue-related experiences may reinforce each other over time (Yao et al., 2025). A fatigue-first ordering is therefore plausible where disengagement is difficult.

Perceived stress captures the broader appraisal that current circumstances exceed available coping resources (Cohen et al., 1983). It has been positively associated with job burnout among nurses, including Chinese samples (Abdollahi et al., 2021; Huang et al., 2025; Tatala et al., 2025). Conservation of resources theory integrates these links (Halbesleben et al., 2014; Hobfoll et al., 2018). The theory proposes that resource depletion heightens sensitivity to further loss and weakens the capacity to manage subsequent demands. On this account, digital fatigue reflects strain from sustained resource expenditure, FoMO reflects concern about what disengagement might forfeit, and perceived stress reflects the broader appraisal that available resources are insufficient. These processes may therefore connect digital fatigue with job burnout separately and sequentially. In this study, FoMO retains its general meaning and is not treated as a measure of fear of missing clinical information.

Guided by conservation of resources theory, the present study aimed to examine the association between digital fatigue and job burnout among nurses in Chinese tertiary hospitals and to test the separate and serial mediating roles of FoMO and perceived stress. Chinese tertiary-hospital nurses constitute a relevant population because policy-driven digitalization has embedded ICT into routine clinical practice, while previous studies have documented both intensive occupational ICT use and a substantial burden of burnout in this group. Theoretically, the study extends demand-focused research on technostress by examining digital fatigue as an experienced form of technology-referent depletion and by integrating general FoMO and perceived stress into a single explanatory model. Practically, identifying these associations may provide a basis for future research on nurses’ digital engagement, stress management, and occupational recovery.

## 2 Literature review and hypothesis development

### 2.1 Conservation of resources theory

Conservation of resources (COR) theory explains psychological strain in terms of the acquisition, protection, and loss of valued resources. Resources include conditions and personal capacities that help individuals attain important goals, as well as energies such as time, attention, and physical or emotional stamina. Resource loss is more salient than equivalent resource gain, and people must invest resources to meet demands, protect what remains, and recover from prior loss. When continued demands leave fewer resources available for further investment or recovery, losses may accumulate in a loss spiral (Halbesleben et al., 2014; Hobfoll, 1989; Hobfoll et al., 2018).

COR theory is particularly relevant to nursing because nurses repeatedly invest limited attentional, emotional, and physical resources in demands that often cannot be deferred. A synthesis of nursing research has framed burnout as a response to threatened or actual losses of energetic, personal, and contextual resources (Prapanjaroensin et al., 2017). Recent findings are consistent with this perspective: ICT demands and psychological stress have been associated with job burnout or emotional exhaustion (Achmet and Lauzier, 2025; Guo et al., 2025), while inclusive leadership and distributive justice have been examined as organizational resources in relation to emotional exhaustion, work engagement, and turnover intention among intensive-care nurses (Zhang et al., 2025). Together, these studies show that resource-relevant demands and conditions covary with consequential nursing outcomes.

COR theory connects the four constructs through their implications for available resources. Digital fatigue reflects the strain experienced during sustained digital engagement. FoMO concerns the possibility of missing valued experiences or connections, while perceived stress captures the appraisal that current demands exceed coping capacity. Job burnout concerns exhaustion and cynicism toward work. Examining these constructs together allows the study to consider how digital fatigue may relate to job burnout directly, through each proposed mediator, and through FoMO and perceived stress in sequence.

### 2.2 Digital fatigue and job burnout

Research on adverse experiences with digital technology employs several related but non-equivalent constructs. Technostress describes a stress process arising from difficulty adapting to or coping with technology and is commonly examined through stressors such as technological overload, invasion, complexity, insecurity, and uncertainty (La Torre et al., 2019). Digital stress often refers to employees’ appraisals of digital working conditions, including communication overload, interruptions, technical problems, and expectations of constant availability (Fischer et al., 2021). Electronic-health-record burden concerns documentation effort, usability problems, and workflow demands associated with a particular clinical system (Alobayli et al., 2023; Wu et al., 2024). Information overload occurs when the volume or complexity of incoming information exceeds the time or capacity available to process it (Arnold et al., 2023). General work fatigue, in contrast, captures physical, mental, or emotional fatigue arising from work without identifying a digital source (Frone and Tidwell, 2015).

Digital fatigue differs from these constructs by focusing on the experience associated with sustained digital engagement rather than on a particular technological demand or system. The Digital Fatigue Scale used in the present study covers difficulty regulating digital engagement, pressure to remain online, psychological and physical fatigue, and psychosomatic discomfort (Tutar and Mutlu, 2024). Digital fatigue is therefore broader than fatigue caused by a single technology, such as an electronic health record or videoconferencing platform, but remains anchored in interaction with digital technologies. Job burnout has a different referent. It concerns an individual’s broader relationship with work and is commonly characterized by exhaustion, psychological distance or cynicism, and reduced professional efficacy (Maslach and Leiter, 2016). Digital fatigue and job burnout may share experiences of fatigue and distress, but they differ in their primary object. One concerns digital engagement, whereas the other concerns the work role as a whole.

Evidence linking digital working conditions with job burnout is substantial. Digital technology stress, electronic-health-record usability problems, techno-overload, techno-complexity, and ICT demands have been associated with emotional exhaustion or burnout among healthcare professionals and nurses (Guo et al., 2025; Li and Zhong, 2026; Melnick et al., 2021; Wirth et al., 2024; Wu et al., 2024). These findings establish the relevance of technology-related demands but do not directly test the broader subjective experience captured by digital fatigue. Using the same Digital Fatigue Scale as the present study, Demir et al. (2025) found that digital fatigue was associated with occupational stress and poorer job performance among hospital nurses, although job burnout was not examined. Outside health care, digital fatigue has been positively associated with burnout and negatively associated with work engagement (Hadjri et al., 2025; Zhang and Deng, 2025).

COR theory connects these findings by treating digital fatigue as a subjective indicator that digital engagement is occurring under strained attentional, energetic, or self-regulatory resources. Because these resources are also required for managing workload, emotional demands, and interpersonal responsibilities, strain associated with digital engagement may extend into nurses’ broader experience of work. When resource restoration is insufficient, nurses reporting greater digital fatigue should therefore also report greater job burnout.

*Hypothesis 1:* Digital fatigue is positively associated with job burnout among Chinese nurses.

### 2.3 The mediating role of fear of missing out

FoMO refers to apprehension about missing rewarding experiences and a desire to remain connected with what others are doing (Przybylski et al., 2013). It has been associated with psychological distress, problematic smartphone use, and compulsive Internet use (Akbari et al., 2021; Elhai et al., 2016; Tandon et al., 2021). Concern about what might be missed may encourage continued monitoring even when digital engagement is tiring.

General FoMO concerns valued experiences, information, and social connections across life domains. Workplace FoMO focuses on occupational information and professional relationships, with informational FoMO referring specifically to work-related updates and knowledge (Budnick et al., 2020; Marsh et al., 2024). The present study measures general FoMO. Workplace studies provide related evidence about connectivity concerns and occupational well-being, while the proposed mediator retains its broader meaning.

Studies of social media have linked FoMO with compulsive use, social comparison, information overload, and fatigue (Dhir et al., 2018; Hattingh et al., 2022; Tandon et al., 2021). Nursing research also establishes its occupational relevance. General FoMO has been associated with perceived work overload and nomophobia among Turkish nurses, job burnout among Omani nurses, and depressive symptoms among Chinese nurses (Hoşgör et al., 2021; Jiang et al., 2025; Qutishat and Al Sabei, 2024). Workplace studies likewise associate FoMO with burnout and difficulties disengaging from work (Budnick et al., 2020; Fridchay and Reizer, 2022; Reimann et al., 2024). These findings suggest that concern about missing out may accompany strain extending beyond digital engagement itself.

The proposed mediating role also requires a rationale for placing digital fatigue before FoMO. Although many studies examine FoMO as an antecedent of fatigue, Yao et al. (2025) found reciprocal associations in a two-wave study of college students: earlier social-media fatigue predicted later FoMO, as well as the reverse. This finding supports considering fatigue as a possible antecedent of connectivity concerns. In nursing, digital engagement is partly required by clinical work and therefore need not originate in FoMO (Guo et al., 2025). Toker et al. (2026) further found that academic nurses described digital fatigue alongside continued online availability and work demands extending into personal time. These accounts show how fatigue can coexist with continued digital engagement.

COR theory offers a rationale for this pattern. When resources are already strained, further losses become more consequential (Halbesleben et al., 2014; Hobfoll et al., 2018). Nurses experiencing digital fatigue may therefore become more concerned about losing access to valued experiences, information, or social connections. Efforts to maintain these connections may require additional attention and limit opportunities to recover, making exhaustion and cynicism toward work more likely. Together with evidence linking FoMO and nurses’ job burnout (Qutishat and Al Sabei, 2024), this argument supports a positive mediating role for FoMO.

*Hypothesis 2:* The association between digital fatigue and job burnout among Chinese nurses is positively mediated by fear of missing out.

### 2.4 The mediating role of perceived stress

Perceived stress refers to an overall appraisal that current circumstances are unpredictable, difficult to control, or excessive relative to available coping resources (Cohen et al., 1983; Lazarus and Folkman, 1984). Digital fatigue reflects depletion associated with sustained digital engagement, whereas perceived stress concerns how manageable current demands feel. This distinction makes perceived stress a plausible mediator: fatigue may matter for job burnout partly because nurses who feel depleted also find their broader responsibilities harder to manage.

Empirical evidence supports a connection between digital fatigue and stress. Demir et al. (2025) found that greater digital fatigue was associated with higher perceived occupational stress among hospital nurses. Qualitative findings provide further context. Academic nurses described difficulties concentrating, continued online availability, and reduced recovery time in their accounts of digital fatigue (Toker et al., 2026). Digital workplace research also links information overload and technology-related demands with stress and exhaustion (Fischer et al., 2021; Marsh et al., 2024). These studies concern occupational or digital stress, while the present study examines the broader appraisal measured by the Perceived Stress Scale.

The second link, between perceived stress and job burnout, is supported by nursing research across several settings. Abdollahi et al. (2021) found that nurses reporting higher perceived stress also reported greater job burnout. Among Chinese neonatal nurses, perceived stress was positively associated with burnout and emotional exhaustion (Huang et al., 2023), and Tatala et al. (2025) reported positive relationships between perceived stress and burnout dimensions. Huang et al. (2025) additionally found that perceived stress mediated the association between anxiety and emotional exhaustion among Chinese infectious-disease nurses. Together, these findings support examining stress appraisal as an intermediate link between an adverse psychological experience and occupational exhaustion.

More closely related evidence comes from intensive-care nursing. Sarmadi et al. (2026) reported a positive indirect association between alarm fatigue and job burnout through perceived stress. Alarm fatigue concerns responses to repeated clinical alarms, while digital fatigue encompasses a wider range of experiences with digital technologies. Their finding provides a nursing-specific precedent for examining perceived stress as a mediator between technology-related fatigue and job burnout. Combined with the digital-fatigue–stress association reported by Demir et al. (2025), it provides an empirical basis for the proposed pathway.

COR theory explains why these links may form a mediated relationship. Attention and energy used during digital engagement are also needed to manage subsequent demands. When nurses feel digitally fatigued, the resources available for coping may seem insufficient, making ongoing responsibilities feel less controllable or manageable (Halbesleben et al., 2014; Hobfoll et al., 2018). Sustained difficulty meeting these demands may be accompanied by emotional exhaustion and cynicism toward work, consistent with resource-based accounts of nursing burnout (Prapanjaroensin et al., 2017). Perceived stress thus represents the appraisal through which digital fatigue may relate to job burnout. This reasoning leads to the following hypothesis.

*Hypothesis 3:* The association between digital fatigue and job burnout among Chinese nurses is positively mediated by perceived stress.

### 2.5 The serial mediating roles of fear of missing out and perceived stress

FoMO and perceived stress may also operate in sequence in the association between digital fatigue and job burnout. The preceding hypotheses address each mediator separately. The serial mediation hypothesis adds the proposition that greater FoMO is associated with greater perceived stress, linking the concern about missing out to job burnout through stress appraisal.

Evidence for this link comes from workplace research. Marsh et al. (2024) found that digital workplace stress mediated the positive association between informational FoMO and exhaustion. Reimann et al. (2025) similarly associated workplace FoMO with higher perceived state stress. In a two-wave study, Ebner et al. (2025) found that increases in relational workplace FoMO were associated with increased perceived stress; the corresponding association for informational FoMO was not significant. The findings show that the content of FoMO matters and provide a basis for examining stress alongside the general FoMO construct assessed here.

COR theory explains the proposed ordering in terms of attempts to protect valued resources. Nurses experiencing digital fatigue may be concerned about what reducing their engagement could cost them. FoMO may encourage them to invest further time and attention in maintaining connections, even when they already feel depleted. Such efforts may make current demands harder to manage when opportunities for recovery are limited (Halbesleben et al., 2014; Hobfoll et al., 2018). Perceived stress captures this broader difficulty in coping. Given its established association with nurses’ job burnout, it may connect FoMO with exhaustion and cynicism toward work (Abdollahi et al., 2021; Huang et al., 2023).

Together, the evidence and COR rationale support the hypothesis that FoMO and perceived stress sequentially mediate the association between digital fatigue and job burnout, with a positive serial mediation effect.

*Hypothesis 4:* The positive association between digital fatigue and job burnout among Chinese nurses is serially mediated by fear of missing out and perceived stress, in that order.

## 3 Method

### 3.1 Design, setting, and data collection

A multicenter cross-sectional survey was conducted from January through May 2026 in five tertiary Grade A hospitals in Hunan Province, China. The hospitals and participating departments were selected through convenience-based institutional collaboration rather than probability sampling. Electronic health records, nursing information systems, mobile nursing terminals, and related digital systems were routinely used in all participating hospitals for clinical documentation, information verification, task execution, and organizational communication.

After authorization was obtained from the nursing administration of each hospital, the research team supplied a standardized invitation and a link to the electronic questionnaire. Head nurses only forwarded the invitation and could not identify participants or access individual responses. The information sheet stated that participation was voluntary, participation or refusal would have no employment consequences, and respondents could discontinue before submission without penalty. The research team did not disclose participation status or individual responses to supervisors. These procedures were intended to reduce perceived participation pressure arising from recruitment through the organizational hierarchy.

Before entering the questionnaire, nurses reviewed an electronic study-information and consent page describing the study purpose, procedures, expected completion time, confidentiality protections, compensation, potential risks, and ethical approval. Only nurses who selected the consent option could proceed. The study was approved by the Academic Board of the School of Journalism and Communication, Chongqing University, on December 29, 2025, and was conducted in accordance with the Declaration of Helsinki and applicable institutional requirements. Respondents received RMB 15 after submitting the questionnaire; compensation was unrelated to response content.

### 3.2 Participants and sample size

Eligible participants were required to (a) hold a valid registered-nurse license; (b) have at least 6 months of direct clinical nursing experience at the current institution and not be completing an internship or standardized training placement; (c) use at least one hospital digital system in routine work; (d) be able to complete an electronic questionnaire independently; and (e) provide informed consent. Nurses on extended leave, no longer engaged in clinical work, or working primarily in nonclinical administrative roles were excluded.

Because the primary analyses involved confirmatory factor analysis, structural equation modeling, and bootstrap tests of indirect associations, the required sample size was determined through a model-specific Monte Carlo analysis (Muthén and Muthén, 1998; Muthén and Muthén, 2002). Before recruitment, an *a priori* simulation was conducted in Mplus 8.3. Population factor loadings were set between 0.50 and 0.80 based on pilot data, and the structural paths and variable distributions were specified according to the hypothesized model. Across 5,000 replications, the minimum sample needed for the least powerful focal path to achieve 0.85 power at *α* = 0.05 was estimated as 364. The recruitment target was increased to 600 completed submissions to improve estimation stability and allow for unusable or duplicate records.

The survey produced 600 complete submissions. Following the prespecified quality-screening procedures described below, 46 records were excluded and 554 were retained for analysis (retention rate = 92.33%). The five participating hospitals contributed 128, 117, 109, 102, and 98 nurses to the final analytic sample. The sample included 516 women (93.14%) and 38 men (6.86%). The predominantly female composition is consistent with the broader sex distribution of China’s nursing workforce (National Health Commission of the People’s Republic of China, 2022). Consistent with comparable nursing studies that retained male participants in predominantly female samples (Jiaqing et al., 2025; Ren et al., 2024), all 38 male nurses who met the study’s eligibility criteria were retained in the present analysis. Sociodemographic and professional information was collected on age, sex, marital status, education, employment status, clinical experience, professional rank, and monthly income. The distributions of these characteristics are presented in Table 1.

**Table 1:** Participant characteristics.

Characteristic	Category	*n*	%
Sex	Men	38	6.86
	Women	516	93.14
Age, years	<30	148	26.71
	30–40	288	51.99
	>40	118	21.30
Marital status	Married	432	77.98
	Unmarried	122	22.02
Education	Associate degree or lower	96	17.33
	Bachelor’s degree or higher	458	82.67
Employment status	Permanent position	126	22.74
	Contract/no permanent position	428	77.26
Clinical experience	<10 years	306	55.23
	10–20 years	200	36.10
	>20 years	48	8.66
Professional rank	Staff nurse or lower	235	42.42
	Senior nurse	189	34.12
	Nurse-in-charge or higher	130	23.47
Monthly income, RMB	<5,000	282	50.90
	5,000–10,000	216	38.99
	>10,000	56	10.11

### 3.3 Measures

#### 3.3.1 Digital fatigue

Digital fatigue was assessed using the 28-item Digital Fatigue Scale (DFS; Tutar and Mutlu, 2024). The scale comprises four dimensions: digital addiction, digital overload and pressure, mental and physical fatigue, and psychosomatic problems. Items were rated from 1 (strongly disagree) to 5 (strongly agree) and averaged, with higher scores indicating greater general digital fatigue. The DFS was translated into Chinese, independently back-translated, reviewed by experts, and culturally adapted for this study. Cronbach’s *α* was 0.874 in the present sample. Because the items assess digital fatigue generally rather than fatigue arising exclusively from hospital systems, the resulting score was not interpreted as a work-specific measure.

#### 3.3.2 Job burnout

Job burnout was measured using a 10-item measure comprising the emotional-exhaustion and cynicism subscales of the Maslach Burnout Inventory–General Survey (MBI-GS). This two-dimensional measurement approach was based on Spence Laschinger and Fida (2014), who assessed nurses’ burnout using five emotional-exhaustion items and five cynicism items, and Guo et al. (2025), who adopted the same two dimensions and item structure among Chinese nurses in a digital healthcare context. In the present study, emotional exhaustion and cynicism were each measured with five items. All items were rated on a five-point scale ranging from 1 to 5, and none were reverse-scored. Mean scores were calculated separately for the two dimensions, and the mean of all 10 items was used as the overall job-burnout score. Higher scores indicated greater job burnout characterized by emotional exhaustion and cynicism. Professional efficacy was not assessed; therefore, job burnout in this study refers specifically to its emotional-exhaustion and cynicism components rather than the complete three-dimensional MBI-GS construct. Cronbach’s *α* was 0.905 for emotional exhaustion, 0.858 for cynicism, and 0.907 for the combined score.

#### 3.3.3 Fear of missing out

FoMO was assessed using the Chinese adaptation of the Fear of Missing Out Scale. The original 10-item scale was developed by Przybylski et al. (2013). The Chinese version validated by Li et al. (2019) contains eight items across two dimensions, fear of missing information and fear of missing social situations. Items are rated from 1 (not at all true of me) to 5 (extremely true of me), with higher scores indicating stronger FoMO. Cronbach’s *α* was 0.783 in the present sample.

#### 3.3.4 Perceived stress

Perceived stress was measured using the 14-item Chinese version of the Perceived Stress Scale (PSS; Cohen et al., 1983; Yang and Huang, 2003). The instrument assesses tension and perceived loss of control. Items were rated from 0 (never) to 4 (very often). After the positively worded items were reverse-scored, the mean of the 14 items was calculated, yielding a score ranging from 0 to 4. Higher scores indicated greater perceived stress. Cronbach’s *α* was 0.851 in the current sample.

### 3.4 Data-quality procedures

Before formal data collection, a pilot survey with 30 nurses who resembled the target population was used to assess item comprehensibility, completion time, platform functionality, and recruitment procedures. The formal survey collected no names or employee identification numbers. All substantive items were required, although respondents could close the survey before submission.

Records were screened using criteria specified before analysis. A record was excluded if (a) completion time was less than 3 min, (b) the embedded attention-check item was answered incorrectly, or (c) technical identifiers together with response information indicated a duplicate submission. The attention-check item stated: “Please select ‘strongly agree’ for this item,” for which the required response was “strongly agree.” Duplicate-submission controls were enabled on Wenjuanxing. Submission times and system-generated identifiers were considered jointly with response patterns; a shared Internet Protocol address alone was not treated as evidence of duplication because hospital networks may use common addresses.

Pilot completion times ranged from 3.5 to 18.2 min. A three-minute cutoff, below the shortest pilot completion time, was prespecified as the screening threshold. Exclusion criteria were applied hierarchically so that each record was counted once. Of the 46 excluded records, 20 were excluded for completion in less than 3 min, 15 for failing the attention check, and 11 as probable duplicate submissions.

### 3.5 Statistical analysis

Descriptive statistics, zero-order Pearson correlations, and internal consistency coefficients (Cronbach’s *α*) were computed in SPSS 26.0. The measurement and structural models were estimated in AMOS 26.0. The CFA and structural models used the 10 dimension scores as observed indicators of the four latent constructs (four for digital fatigue and two each for job burnout, FoMO, and perceived stress), a specification that kept the number of estimated parameters appropriate for the sample size. Potential common method variance was examined initially using an unrotated Harman single-factor diagnostic, while recognizing that this procedure cannot rule out common method bias (Podsakoff et al., 2003).

A confirmatory factor analysis compared the hypothesized four-construct measurement model with three-, two-, and one-factor alternatives. Model fit was evaluated using *χ^2^*, the *χ^2^*/df ratio, the comparative fit index (CFI), Tucker-Lewis index (TLI), goodness-of-fit index (GFI), root mean square error of approximation (RMSEA), and standardized root mean square residual (SRMR). Variance inflation factors (VIFs) were inspected as a supplementary check of predictor collinearity.

The hypothesized structural model specified paths from digital fatigue to FoMO, perceived stress, and burnout; from FoMO to perceived stress and burnout; and from perceived stress to burnout. Specific indirect associations were evaluated using 5,000 bias-corrected bootstrap resamples. An indirect association was considered statistically significant when its 95% confidence interval did not include zero. Because the design was cross-sectional, the estimated paths were interpreted as theoretically ordered associations rather than evidence of temporal or causal mediation.

The unadjusted model (Model 0) was used for the primary hypothesis tests. Age, sex, and employment status were included as covariates in the primary adjusted sensitivity model (Model 1), informed by previous evidence linking demographic and employment characteristics with dimensions of nurses’ job burnout (Li et al., 2024a). Model 2 replaced age with clinical experience to examine an alternative representation of professional seniority. Model 3 included all eight sociodemographic and professional variables reported in Table 1. Categorical covariates were dummy-coded. In each adjusted model, covariates were entered as exogenous predictors of FoMO, perceived stress, and job burnout and were allowed to covary with digital fatigue. Indirect associations were re-estimated using 5,000 bias-corrected bootstrap resamples.

## 4 Results

### 4.1 Common method variance diagnostic

Because all focal variables were assessed using self-report measures collected at a single time point, common method variance was evaluated before testing the hypothesized model. All 60 measurement items were entered into an unrotated exploratory factor analysis (Harman’s single-factor test). Seven factors with eigenvalues greater than 1 were extracted, and the first factor accounted for 24.81% of the total variance. No single factor accounted for the majority of item variance, providing preliminary evidence that the observed covariance structure was unlikely to be dominated by a single common method factor.

This result should nevertheless be interpreted cautiously. Harman’s single-factor test has limited sensitivity and cannot establish the absence of common method bias. Moreover, because the four instruments were designed to assess multidimensional substantive constructs, the extracted factors may reflect both substantive scale structure and shared measurement characteristics. Accordingly, the test was treated as a diagnostic procedure rather than definitive evidence against common method variance. The confirmatory factor analysis reported below provided an additional assessment of whether digital fatigue, FoMO, perceived stress, and job burnout could be empirically distinguished.

### 4.2 Measurement model

A confirmatory factor analysis was conducted to evaluate the measurement model comprising four correlated latent constructs: digital fatigue, FoMO, perceived stress, and job burnout. The hypothesized four-construct model demonstrated an acceptable overall fit to the data, *χ^2^*/df = 2.65, RMSEA = 0.055, CFI = 0.948, TLI = 0.937, SRMR = 0.052, and GFI = 0.942. The *χ^2^*/df and RMSEA values were below conventional upper limits of 3 and 0.08, respectively, and GFI exceeded 0.90 (Schermelleh-Engel et al., 2003). CFI and TLI approached the more stringent 0.95 criterion, and SRMR was below 0.08 (Hu and Bentler, 1999). Taken together, these indices indicated that the proposed measurement structure provided an adequate representation of the observed data.

As reported in Table 2, the four-construct model fit the data better than the three-, two-, and single-factor alternatives, and fit deteriorated progressively as theoretically distinct constructs were combined (ΔCFI = 0.027–0.124). The largest deterioration occurred when all indicators were forced onto a single factor. These comparisons support the empirical distinctiveness of digital fatigue, FoMO, perceived stress, and job burnout. Because the measurement model was specified at the dimension level, standardized loadings of the dimension scores on their latent constructs ranged from 0.66 to 0.87, and no correlated residual terms were specified. The absolute standardized residual covariances ranged from 0.02 to 2.14, and no standardized residual exceeded 2.58.

**Table 2:** Confirmatory factor analysis.

Model	*χ^2^*	df	*χ^2^*/df	RMSEA	CFI	TLI	GFI	SRMR	ΔCFI
Four-factor model	76.93	29	2.653	0.055	0.948	0.937	0.942	0.052	–
Three-factor model	112.71	32	3.522	0.068	0.921	0.904	0.918	0.058	0.027
Two-factor model	150.88	34	4.438	0.079	0.883	0.858	0.886	0.072	0.065
Single-factor model	207.9	35	5.940	0.095	0.824	0.789	0.831	0.089	0.124

*N* = 554. All *χ^2^* values were statistically significant (*p* < 0.001). RMSEA 90% confidence intervals were [0.040, 0.070] for the four-factor model, [0.054, 0.081] for the three-factor model, [0.066, 0.092] for the two-factor model, and [0.082, 0.107] for the single-factor model. The three-factor model combined the indicators of digital fatigue and job burnout into one factor; the two-factor model additionally combined the indicators of FoMO and perceived stress; the single-factor model loaded all indicators on one factor. ΔCFI = change in CFI relative to the four-factor model. RMSEA = root mean square error of approximation; CFI = comparative fit index; TLI = Tucker-Lewis index; GFI = goodness-of-fit index; SRMR = standardized root mean square residual.

Potential multicollinearity among the observed predictor scores was additionally examined using variance inflation factors. VIF values ranged from 1.19 to 1.21, substantially below conventional thresholds for problematic collinearity. Thus, the estimated structural paths were unlikely to have been materially distorted by excessive linear dependence among digital fatigue, FoMO, and perceived stress.

### 4.3 Descriptive statistics and correlations

Means, standard deviations, internal consistency coefficients, and Pearson correlations for the focal variables are presented in Table 3. Digital fatigue was positively correlated with job burnout, *r* = 0.417, *p* < 0.001, indicating that nurses reporting greater fatigue associated with digital engagement also tended to report higher levels of burnout. Digital fatigue was also positively correlated with FoMO, *r* = 0.352, *p* < 0.001, and perceived stress, *r* = 0.326, *p* < 0.001.

**Table 3:** Descriptive statistics, reliability, and correlations.

Variable	*M*	SD	*α*	Digital fatigue	Job burnout	FoMO	Perceived stress
Digital fatigue	3.54	0.68	0.874				
Job burnout	2.92	0.81	0.907	0.417^***^			
Fear of missing out	2.87	0.73	0.783	0.352^***^	0.273^***^		
Perceived stress	2.66	0.59	0.851	0.326^***^	0.538^***^	0.328^***^	

*α* = Cronbach’s alpha coefficient; FoMO = fear of missing out. ^*^p < 0.05, ^**^p < 0.01, ^***^p < 0.001.

The two proposed intermediate variables were likewise associated with job burnout. FoMO was positively correlated with job burnout, *r* = 0.273, *p* < 0.001, whereas perceived stress showed a stronger positive correlation with job burnout, *r* = 0.538, *p* < 0.001. FoMO was also positively correlated with perceived stress, *r* = 0.328, *p* < 0.001. Thus, nurses reporting greater general concern about missing rewarding experiences or social connection also tended to perceive their circumstances as more unpredictable, uncontrollable, or overwhelming.

All correlations were in the hypothesized directions. Because these bivariate associations did not account for overlap among variables, the direct and specific indirect associations were evaluated in the structural model.

### 4.4 Mediated pathways

Based on the conceptual model, a structural equation model was specified with digital fatigue as the predictor, job burnout as the outcome, and FoMO and perceived stress as parallel and sequential intermediate variables. The model included direct paths from digital fatigue to FoMO, perceived stress, and job burnout; paths from FoMO to perceived stress and job burnout; and a path from perceived stress to job burnout. The unstandardized and standardized estimates are reported in Table 4 and illustrated in Figure 1. Because all six recursive paths implied by the prespecified ordering were freely estimated, the latent structural component was saturated (df = 0). Consequently, with the measurement specification unchanged, the full SEM produced the same global fit indices as the CFA: *χ^2^*/df = 2.65, RMSEA = 0.055 (90% CI [0.040, 0.070]), CFI = 0.948, TLI = 0.937, and SRMR = 0.052. These global indices therefore did not distinguish the proposed directional ordering from alternative saturated recursive orderings.

**Table 4:** Structural path estimates.

Path	SE	*β*	CR	*p*	95% CI
Digital fatigue → FoMO	0.057	0.352	6.632	<0.001	[0.266, 0.490]
Digital fatigue → Perceived stress	0.055	0.158	2.691	0.007	[0.040, 0.256]
Digital fatigue → Job burnout	0.049	0.218	3.959	<0.001	[0.098, 0.290]
FoMO → Perceived stress	0.049	0.270	4.367	<0.001	[0.118, 0.310]
FoMO → Job burnout	0.045	0.121	2.578	0.010	[0.028, 0.204]
Perceived stress → Job burnout	0.055	0.427	8.218	<0.001	[0.344, 0.560]

*β* = standardized coefficient. The 95% confidence intervals were computed as *B* ± 1.96 × SE.

**Figure 1:**
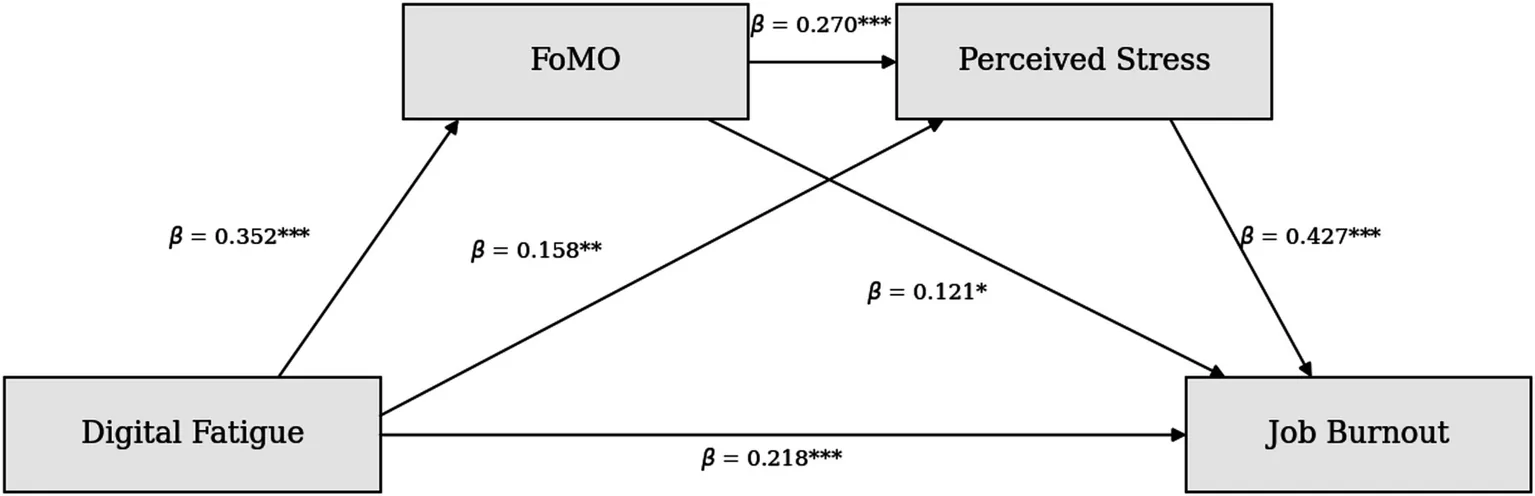
Structural equation model path coefficients.

All six specified structural paths were statistically significant. Digital fatigue was positively associated with FoMO (*β* = 0.352, *p* < 0.001), perceived stress (*β* = 0.158, *p* = 0.007), and job burnout (*β* = 0.218, *p* < 0.001). FoMO was positively associated with perceived stress (*β* = 0.270, *p* < 0.001) and job burnout (*β* = 0.121, *p* = 0.010), while perceived stress was positively associated with job burnout (*β* = 0.427, *p* < 0.001). Perceived stress showed the strongest direct association with job burnout. The model explained 12.4% of the variance in FoMO, 12.8% of the variance in perceived stress, and 34.4% of the variance in job burnout.

Specific indirect associations were examined using bias-corrected bootstrap confidence intervals based on 5,000 resamples. As shown in Table 5, the indirect association between digital fatigue and job burnout through FoMO was 0.043 (95% CI [0.015, 0.078]), the indirect association through perceived stress was 0.067 (95% CI [0.023, 0.114]), and the serial indirect association through FoMO followed by perceived stress was 0.041 (95% CI [0.021, 0.066]). None of the confidence intervals included zero. The total standardized indirect association was 0.151. The indirect associations together represented 40.9% of the total association (0.151/0.369), whereas the remaining direct association represented 59.1% (0.218/0.369). Bootstrap standard errors and confidence intervals for the direct, total indirect, and total associations are reported in Table 5.

**Table 5:** Direct, indirect, and total associations.

Path	Standardized estimate	Bootstrap SE	Bias-corrected 95% CI
Digital fatigue → FoMO → Job burnout	0.043	0.016	[0.015, 0.078]
Digital fatigue → Perceived stress → Job burnout	0.067	0.023	[0.023, 0.114]
Digital fatigue → FoMO → Perceived stress → Job burnout	0.041	0.011	[0.021, 0.066]
Total indirect association	0.151	0.038	[0.080, 0.228]
Direct association (digital fatigue → job burnout)	0.218	0.055	[0.112, 0.328]
Total association	0.369	0.067	[0.240, 0.503]

Indirect associations were estimated using 5,000 bias-corrected bootstrap resamples, and inference was based on the bootstrap confidence intervals rather than normal-theory *z* tests. The total indirect association is the sum of the three specific indirect associations, and the total association is the sum of the direct and total indirect associations; all were computed from unrounded coefficients.

### 4.5 Sensitivity analyses

To evaluate the robustness of the structural model, the model was re-estimated under three alternative covariate specifications (Table 6). Model 0 served as the unadjusted reference model, and the hypothesis tests reported in Section 4.4 were based on this unadjusted specification. Model 1, adjusted for age, sex, and employment status, was the primary adjusted model. Model 2 replaced age with clinical experience to examine whether the estimates depended on the operationalization of professional seniority. Model 3 simultaneously included all measured demographic variables, namely age, sex, marital status, education, employment status, clinical experience, professional rank, and monthly income, with categorical variables dummy-coded. In each adjusted specification, the covariates were entered as exogenous predictors of FoMO, perceived stress, and job burnout and were allowed to covary with digital fatigue. All indirect associations were re-estimated using 5,000 bias-corrected bootstrap resamples.

**Table 6:** Sensitivity analyses across alternative covariate specifications.

Model	DF → FoMO	DF → PS	FoMO → PS	DF → JB	FoMO → JB	PS → JB	Total indirect
Model 0: unadjusted	0.352	0.158	0.270	0.218	0.121	0.427	0.151
Model 1: demographic adjustment	0.337	0.163	0.276	0.209	0.114	0.420	0.145
Model 2: clinical experience replaces age	0.348	0.155	0.265	0.215	0.125	0.432	0.150
Model 3: all measured demographics	0.325	0.168	0.283	0.202	0.108	0.438	0.149

All values are standardized estimates. Model 1 adjusted for age, sex, and employment status; Model 2 adjusted for clinical experience, sex, and employment status; Model 3 included age, sex, marital status, education, employment status, clinical experience, professional rank, and monthly income, with categorical variables dummy-coded. The total indirect association is the sum of the three specific indirect associations and was computed from unrounded coefficients. All path coefficients were statistically significant (*p* < 0.05). DF = digital fatigue; PS = perceived stress; JB = job burnout.

Sensitivity analyses yielded generally comparable estimates across the alternative covariate specifications (Table 6). Relative to the unadjusted model, absolute changes in the six focal path coefficients ranged from 0.003 to 0.027, and the rank ordering of the six paths was identical in every specification. The largest change was observed for the association between digital fatigue and FoMO in the fully adjusted model (*β* = 0.325 vs. 0.352), a relative attenuation of 7.7%. The direct association between digital fatigue and job burnout ranged from 0.202 to 0.218, corresponding to a maximum attenuation of 7.3% under full demographic adjustment. All six paths remained positive and statistically significant in every specification (all *p* < 0.05), and global fit indices changed modestly across specifications (*χ^2^*/df < 2.70, CFI = 0.934 to 0.948, TLI = 0.919 to 0.937, RMSEA < 0.06, SRMR = 0.052 to 0.063).

The three specific indirect associations were likewise stable (Table 7). The indirect association through FoMO ranged from 0.035 to 0.044, the association through perceived stress ranged from 0.067 to 0.074, and the serial indirect association ranged from 0.039 to 0.041. Every bias-corrected bootstrap 95% confidence interval excluded zero across all four specifications. The total indirect association ranged from 0.145 to 0.151 and accounted for 40.9 to 42.5% of the total association between digital fatigue and job burnout. The relative ordering of the three indirect components was preserved in every model, with the component operating through perceived stress consistently the largest.

**Table 7:** Specific indirect associations across covariate specifications.

Model	Indirect association	*β*	SE	95% CI
Model 0	Digital fatigue → FoMO → Job burnout	0.043	0.016	[0.015, 0.078]
	Digital fatigue → Perceived stress → Job burnout	0.067	0.023	[0.023, 0.114]
	Digital fatigue → FoMO → Perceived stress → Job burnout	0.041	0.011	[0.021, 0.066]
Model 1	Digital fatigue → FoMO → Job burnout	0.038	0.014	[0.011, 0.067]
	Digital fatigue → Perceived stress → Job burnout	0.068	0.023	[0.023, 0.115]
	Digital fatigue → FoMO → Perceived stress → Job burnout	0.039	0.011	[0.018, 0.061]
Model 2	Digital fatigue → FoMO → Job burnout	0.044	0.016	[0.016, 0.080]
	Digital fatigue → Perceived stress → Job burnout	0.067	0.023	[0.023, 0.114]
	Digital fatigue → FoMO → Perceived stress → Job burnout	0.040	0.011	[0.020, 0.064]
Model 3	Digital fatigue → FoMO → Job burnout	0.035	0.014	[0.009, 0.064]
	Digital fatigue → Perceived stress → Job burnout	0.074	0.025	[0.028, 0.125]
	Digital fatigue → FoMO → Perceived stress → Job burnout	0.040	0.011	[0.020, 0.065]

*β* = standardized indirect association; SE and bias-corrected bootstrap 95% confidence intervals were based on 5,000 resamples.

Across all four models, the indirect association through perceived stress had the largest point estimate among the three specific indirect associations. The estimates therefore showed limited sensitivity to the measured demographic covariates. Because occupational variables such as department, night shifts, working hours, workload, nurse-to-patient ratio, patient acuity, sleep quality, digital competence, organizational support, system usability, and after-hours connectivity were not measured, residual confounding cannot be excluded.

## 5 Discussion

### 5.1 Overview of the main findings

This study examined the association between digital fatigue and job burnout among 554 nurses from five tertiary hospitals in China, with FoMO and perceived stress considered as parallel and sequential intermediate variables. Digital fatigue remained positively associated with job burnout after both intermediate variables were included. The model also identified specific indirect associations through FoMO and perceived stress, together with a serial indirect association through FoMO followed by perceived stress. Perceived stress accounted for the largest specific indirect estimate, although the indirect effects were not formally compared. The model explained 34.4% of the variance in job burnout, and the focal paths changed little across alternative demographic covariate specifications. The findings therefore reveal a differentiated pattern of contemporaneous associations between digital fatigue and burnout.

### 5.2 Digital fatigue and job burnout

Digital fatigue retained a direct association with job burnout after FoMO and perceived stress were considered simultaneously. Qualitative evidence from Turkish academic nurses portrays digital fatigue as a multidimensional experience involving physical discomfort, cognitive disruption, emotional strain, social disconnection, and difficulty maintaining work-life boundaries under constant online connectivity (Toker et al., 2026). Among Turkish hospital nurses, digital fatigue has also been associated with poorer job performance (Demir et al., 2025). The present study extends this evidence to job burnout, operationalized as emotional exhaustion and cynicism, among clinical nurses.

Research using adjacent digital-work constructs shows a similar pattern. ICT demands have been associated with burnout among Chinese nurses (Guo et al., 2025), while techno-overload and techno-complexity have been associated with burnout among German hospital nurses (Wirth et al., 2024). Studies of Malaysian health professionals and Egyptian critical care nurses likewise connect technostress or technology-related demands with adverse occupational outcomes (Muhamad et al., 2025; Shaban et al., 2025). These studies focus mainly on technological conditions or employees’ appraisal of those conditions. Digital fatigue adds the experienced depletion that accompanies sustained digital engagement. Its remaining direct association with burnout suggests that this depletion has occupational relevance beyond its overlap with FoMO and perceived stress.

The Chinese tertiary-hospital setting gives this association practical salience. Electronic documentation, mobile terminals, care-coordination systems, and work-related communication were routinely used across the participating hospitals. The findings therefore establish the relevance of digital fatigue to burnout in a setting where digital systems are embedded in clinical workflows, while the one-province convenience sample limits broader national or cross-country inference.

### 5.3 The specific indirect association through fear of missing out

FoMO accounted for a specific indirect association between digital fatigue and job burnout. In an Omani nursing sample, general FoMO was positively associated with burnout (Qutishat and Al Sabei, 2024). Workplace-FoMO research has also related concern about missing work information or opportunities to checking, leisure-time ICT use, impaired detachment, and poorer well-being (Budnick et al., 2020; Marsh et al., 2024; Reimann et al., 2024). The present results add that nurses who reported greater digital fatigue also tended to report greater general FoMO, and greater FoMO was associated with greater job burnout.

Digital fatigue and FoMO capture different aspects of continued digital engagement: one concerns depletion, whereas the other concerns what might be missed through disconnection. Their positive association is consistent with longitudinal evidence that FoMO and social-media fatigue may reinforce one another over time (Yao et al., 2025). Occupational studies show analogous links between workplace FoMO, continued checking, impaired detachment, work–family conflict, and burnout (Deng et al., 2026; Reimann et al., 2024; Shi et al., 2024). Because the present study assessed general FoMO, its indirect association should be understood in relation to rewarding experiences and social connection broadly, rather than specifically to clinical information.

### 5.4 The specific indirect association through perceived stress

Digital fatigue also had an indirect association with burnout through perceived stress. This pattern indicates that fatigue associated with digital engagement covaried with nurses’ broader appraisal of how manageable their circumstances were. Previous studies have linked digital fatigue with occupational stress among nurses and psychological stress with emotional exhaustion or burnout in nursing samples (Abdollahi et al., 2021; Achmet and Lauzier, 2025; Demir et al., 2025; Huang et al., 2023). Longitudinal nursing research further shows that work stress, burnout, job satisfaction, and health remain interrelated over time (Khamisa et al., 2016). Together, this evidence supports treating digital fatigue, perceived stress, and burnout as related but distinct experiences.

Perceived stress reflects whether circumstances are experienced as uncontrollable, unpredictable, or beyond available coping resources (Cohen et al., 1983; Lazarus and Folkman, 1984). Digital-work research identifies information overload, interruptions, telepressure, and expectations of rapid response as conditions associated with reduced control and impaired recovery (Barber and Santuzzi, 2015; Fischer et al., 2021; Marsh et al., 2024). In the present study, greater digital fatigue was associated with greater perceived stress, which in turn was strongly associated with job burnout. Perceived stress therefore identifies a general appraisal component in the relationship between digital fatigue and burnout.

### 5.5 The serial indirect association through FoMO and perceived stress

The serial indirect association brings FoMO and perceived stress together in the positive relationship between digital fatigue and job burnout. Nurses reporting greater digital fatigue also reported greater FoMO; greater FoMO was associated with greater perceived stress; and greater perceived stress was associated with greater emotional exhaustion and cynicism. Considered together, these associations distinguish fatigue related to digital engagement, concern about missing out, general stress appraisal, and job burnout rather than treating them as a single undifferentiated form of distress.

The closest comparable evidence comes from longitudinal social-media research. In a three-wave study of Chinese college students, perceived stress formed an indirect link between FoMO and social-media fatigue, both independently and within a serial pathway involving information overload (Yao et al., 2026). A separate two-wave study found reciprocal associations: earlier FoMO predicted later social-media fatigue, and earlier fatigue predicted later FoMO (Yao et al., 2025). Although the population, outcome, and ordering differ from the present study, these findings show that FoMO, fatigue, and perceived stress can be linked across time. The present results identify a related cross-sectional pattern with job burnout as the occupational outcome among clinical nurses.

Occupational evidence helps interpret the FoMO–stress segment of this pattern. Informational FoMO has been associated with digital workplace stress and exhaustion (Marsh et al., 2024), while workplace FoMO has been related to leisure-time ICT use, perceived stress, and poorer psychological detachment (Reimann et al., 2024). In nursing, constant online availability and blurred work boundaries have been described alongside reduced recovery (Toker et al., 2026), and after-hours work connectivity has been associated with occupational fatigue through reduced psychological detachment (Qu et al., 2026). Psychological stress, in turn, has been closely associated with emotional exhaustion among nursing managers (Achmet and Lauzier, 2025). COR theory offers a concise account of these linked experiences: concern about missing out may keep attention engaged when resources are already strained, while restricted recovery is associated with broader stress and burnout.

### 5.6 Theoretical contributions and the explanatory scope of COR theory

The first contribution is to shift attention from digital exposure and technology characteristics to the depletion nurses experience. Much of the nursing literature explains burnout through techno-overload, complexity, ICT demands, or electronic-record burden. These conditions describe what the digital environment requires; digital fatigue describes how sustained digital engagement is experienced. By linking digital fatigue with job burnout, the present study identifies an association between technology-referent depletion and a broader occupational outcome. The second contribution is to differentiate the intermediate variables. FoMO concerns anticipated loss through disconnection, whereas perceived stress concerns whether current demands exceed available coping resources. Their simultaneous and serial involvement indicates that connectivity concern and global stress appraisal make distinct contributions to the digital fatigue–job burnout association.

COR theory provides a coherent organizing framework for these findings. People seek to retain valued resources, actual and threatened losses are stressful, and resource loss can increase vulnerability to further loss (Halbesleben et al., 2014; Hobfoll et al., 2018). Digital fatigue is consistent with strain on attention, energy, and self-regulatory capacity; FoMO with sensitivity to possible loss through disconnection; perceived stress with a broader judgment that coping resources are insufficient; and job burnout with strain at the level of the work role. COR theory thus explains why these experiences may cluster, although construct-specific evidence and longitudinal data are needed to establish their ordering and time scale.

### 5.7 Practical and managerial implications

The findings suggest that hospitals could evaluate digitalization not only by efficiency, compliance, and information availability, but also by whether digital work is associated with excessive depletion. Digital fatigue could be included as an occupational-health indicator when digital systems are introduced or reviewed, together with perceived stress, job burnout, and opportunities for recovery. The roles of FoMO and perceived stress also suggest that reducing screen time alone may not address continued psychological connection. Managers could examine communication expectations, message urgency, interruptions, and after-hours availability when assessing nurses’ digital work. The remaining direct association between digital fatigue and burnout also argues against placing responsibility solely on individual nurses through resilience or stress-management programs.

The present study did not test specific organizational interventions. Documentation redesign, alert management, clearer response expectations, protected recovery time, and improved system usability should therefore be treated as candidate strategies for evaluation. Their effects should be examined prospectively by assessing digital fatigue, stress, burnout, workflow, and patient-safety outcomes before and after implementation. The practical contribution of this study is thus to identify digital depletion and continued psychological connectivity as occupational concerns that should be incorporated into the design and evaluation of hospital digital work.

## 6 Limitations and directions for future research

Several limitations define the scope of the findings. First, all focal variables were measured at one time point, so temporal precedence and causal mediation cannot be inferred, and the saturated model cannot identify the best ordering from global fit. Multiwave research should compare proposed, reversed, and reciprocal relations. Intensive longitudinal designs could examine short-term variation in fatigue, FoMO, and stress, whereas longer follow-up would be needed for burnout.

Second, all focal measures were self-reported in the same survey. The four-factor model’s superiority over the tested collapsed alternatives does not eliminate common-method variance or establish cross-cultural invariance. Digital fatigue and FoMO were assessed generally rather than in relation to work or clinical information, perceived stress was global, and burnout comprised exhaustion and cynicism rather than all three MBI-GS dimensions. Future studies should further validate the translated digital-fatigue measure and combine self-reports with system-use, exposure, and recovery indicators.

Third, convenience recruitment from five tertiary Grade A hospitals in one province limits generalizability. The sample included both women and men but was predominantly female, and the small number of male participants limited sex-specific inference. Larger, more diverse multisite samples are needed to test institutional variation and comparability across demographic and national groups.

Finally, residual confounding and unmeasured contextual processes remain possible. The analyses did not include workload, shifts, patient acuity, staffing, digital competence, system usability, sleep, mental health, organizational support, or after-hours connectivity. Nor did they assess whether digital tasks were mandatory, time-sensitive, safety-critical, or difficult to suspend. Future work should measure these conditions, distinguish confounders from pathway variables, and test through intervention studies whether modifying them alters digital fatigue, stress, or burnout.

## 7 Conclusion

Among nurses in five Chinese tertiary hospitals, greater digital fatigue was associated with greater job burnout, operationalized as emotional exhaustion and cynicism. The association included a remaining direct component and specific indirect components through general FoMO and perceived stress, both separately and in sequence. These findings distinguish technology-related depletion, general concern about missing out, global stress appraisal, and burnout while showing that they covary in a theoretically coherent pattern. Longitudinal and intervention research should test competing directions and the effects of modifiable work conditions.

## Data Availability

The original contributions presented in the study are included in the article/supplementary material, further inquiries can be directed to the corresponding author.
